# Effects of Biodiversity Loss on Freshwater Ecosystem Functions Increase With the Number of Stressors

**DOI:** 10.1111/gcb.70617

**Published:** 2025-11-26

**Authors:** Ralf B. Schäfer, Daria Baikova, Helena S. Bayat, Arne J. Beermann, Stella A. Berger, Jens Boenigk, Mario Brauns, Andrea Burfeid‐Castellanos, Bradley J. Cardinale, Gwendoline M. David, Alexander Feckler, Christian K. Feld, Patrick Fink, Mark O. Gessner, Una Hadziomerovic, Daniel Hering, T. T. Yen Le, Samuel J. Macaulay, Graciela Medina Madariaga, Ntambwe A. Serge Mayombo, Iris Madge Pimentel, James A. Orr, Stephen Osakpolor, Alexandra Schlenker, Bernd Sures, Anna‐Maria Vermiert, Matthijs Vos, Markus Weitere, Christian Schürings

**Affiliations:** ^1^ Department of Ecotoxicology and Centre for Water and Environmental Research, Faculty of Biology University of Duisburg‐Essen Essen Germany; ^2^ Research Center One Health Ruhr University Alliance Ruhr, University of Duisburg‐Essen Essen Germany; ^3^ Department of Aquatic Microbiology, Environmental Microbiology and Biotechnology and Centre for Water and Environmental Research, Faculty of Chemistry University of Duisburg‐Essen Essen Germany; ^4^ Department of Aquatic Ecosystem Research and Centre for Water and Environmental Research, Faculty of Biology University of Duisburg‐Essen Essen Germany; ^5^ Department of Plankton and Microbial Ecology Leibniz Institute of Freshwater Ecology and Inland Fisheries (IGB) Stechlin Germany; ^6^ Department of Biodiversity and Centre for Water and Environmental Research, Faculty of Biology University of Duisburg‐Essen Essen Germany; ^7^ Department of River Ecology Helmholtz Centre for Environmental Research – UFZ Magdeburg Germany; ^8^ Department of Phycology and Centre for Water and Environmental Research, Faculty of Biology University of Duisburg‐Essen Essen Germany; ^9^ Department of Ecosystem Science and Management Penn State University Pennsylvania USA; ^10^ Laboratoire Image, Ville, Environnement, UMR LIVE CNRS Université de Strasbourg Strasbourg France; ^11^ Ecole Nationale du Génie de l’Eau et de l’Environnement (ENGEES) Strasbourg France; ^12^ iES Landau, Institute for Environmental Sciences RPTU University of Kaiserslautern‐Landau Landau in der Pfalz Germany; ^13^ Department of Aquatic Ecology and Centre for Water and Environmental Research, Faculty of Biology University of Duisburg‐Essen Essen Germany; ^14^ Department Aquatic Ecosystem Analysis and Management Helmholtz‐Centre for Environmental Research – UFZ Magdeburg Germany; ^15^ Institute for Zoology University of Cologne Cologne Germany; ^16^ Institute of Ecology Berlin Institute of Technology (TU Berlin) Berlin Germany; ^17^ Department of Environmental Science and Technology Hanoi University of Science and Technology Hanoi Vietnam; ^18^ Department of Biology University of Oxford Oxford UK; ^19^ Department of Life Sciences Imperial College London London UK; ^20^ Department of Community and Ecosystem Ecology Leibniz Institute of Freshwater Ecology and Inland Fisheries (IGB) Berlin Germany; ^21^ School of the Environment University of Queensland Brisbane Queensland Australia; ^22^ Department of Animal Ecology, Faculty of Biology and Biotechnology Ruhr University Bochum Bochum Germany; ^23^ Theoretical and Applied Biodiversity Research, Faculty of Biology and Biotechnology Ruhr University Bochum Bochum Germany; ^24^ School of Aquatic and Fishery Sciences University of Washington Seattle Washington USA

**Keywords:** communities, ecosystem processes, environmental drivers, global change, leaf decomposition, litter breakdown, multiple stressors, productivity, rivers

## Abstract

A multitude of anthropogenic stressors drive biodiversity loss and alter ecosystem functioning. Freshwaters, which contribute disproportionally to global biodiversity and biogeochemical cycles, are particularly threatened. Although the relationship between biodiversity and ecosystem functions (BEF) is generally well‐established, especially in terrestrial ecosystems, the role of multiple, co‐occurring stressors in modulating the relationship remains unclear. We conducted a meta‐analysis to address this knowledge gap by assessing the effect of multiple stressors on the relationship between taxon richness and four measures of ecosystem function. The relationship was generally positive, with the slope becoming steeper as the number of stressors increased, suggesting that exposure to multiple stressors exacerbates impacts of biodiversity loss on ecosystem function. Multiple stressor effects on both taxon richness and ecosystem functions were largely predictable from individual stressor effects, although antagonistic effects on ecosystem functions emerged in 14% of the considered cases. The type of stressor and ecosystem function, along with taxonomic group, exerted no influence on the BEF relationship, contrary to our expectations. Microbial production and biomass declined most strongly in response to stressors, despite notable variability. Overall, our findings imply that functional consequences of freshwater biodiversity loss are more severe under multifaceted environmental change than previously assumed.

## Graphical abstract

The paper reports results of a meta‐analysis addressing the effect of multiple stressors on the relationship between biodiversity and four measures of ecosystem function in freshwater ecosystems. We found that the positive relationship between biodiversity and ecosystem functions becomes stronger as the number of stressors increases, indicating that multiple stressors exacerbate declines in ecosystem functioning. Multiple‐stressor effects on the biodiversity‐ecosystem function relationship were largely predictable from single‐stressor impacts, with antagonistic interactions occurring in only 14% of the cases and synergistic effects not being detected at all.

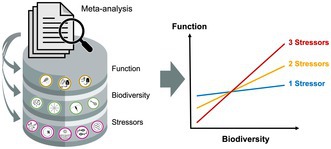

## 1 Introduction

Freshwater ecosystems play a disproportionate role for global biodiversity and biogeochemical processes relative to their small areal extent (Aufdenkampe et al. 2011; Battin et al. 2023; Pekel et al. 2016). Covering only 2.3% of the global land surface area, freshwaters host at least 9.5% of the currently described animal species and remove 20% of all land‐based nitrogen sources (Mulholland et al. 2008; Reid et al. 2019; Seitzinger et al. 2006). They transport and partially process quantities of carbon equivalent to approximately 25% of the estimated global terrestrial net ecosystem production (Battin et al. 2023).

Freshwater ecosystems are affected by a wide range of stressors, defined here as anthropogenic factors that push ecosystems beyond their natural range of variation. Major stressors include habitat degradation through hydromorphological or land use changes, resource exploitation (e.g., mining, fishing), pollution (e.g., nutrient and toxicant inputs), climate change and invasive species (Díaz et al. 2019; Dudgeon et al. 2006; Jaureguiberry et al. 2022). These stressors often co‐occur and can interact to produce non‐additive impacts on ecosystems (Lemm et al. 2021; Schäfer et al. 2016). These impacts have been associated with a turnover in species composition and an overall loss of biodiversity (Ceballos et al. 2015; Cowie et al. 2022; Dornelas et al. 2019; Toszogyova et al. 2024). Consequently, populations of around half of the fish and invertebrate species in streams and rivers are in decline, with most of these impaired as occurrences distinctly deviate from undisturbed conditions (Feio et al. 2023). This widespread impairment is also linked to changes in ecosystem functions, which pertain to processes that control the flow of energy and matter within ecosystems. A global synthesis found that anthropogenic stressors, particularly those related to wastewater discharge, agriculture and urbanisation, strongly affect river ecosystem functions such as organic matter decomposition and biomass production (Brauns et al. 2022). However, the role of biodiversity in these effects was not addressed, though essential for predicting the consequences of biodiversity loss on ecosystem functioning.

Broad evidence from different types of ecosystems supports the hypothesis that biodiversity loss contributes to reduced ecosystem functions (Artamendi et al. 2025; Cardinale et al. 2012; Hong et al. 2022; Tilman et al. 2014). Further, this relationship between biodiversity loss and ecosystem functions is typically negative and amplifying. However, the shape can vary with ecosystem type, ecosystem process, organism group, the degree of overlap in functional traits among community members, and spatial and temporal scales at which the relationship is examined (Cardinale et al. 2012; Hooper et al. 2012; Qiu and Cardinale 2020; Smeti et al. 2019). The type of stressor (e.g., a toxicant or climate change) may also be influential (Beaumelle et al. 2020; Jonsson et al. 2002). For example, the results of a meta‐analysis suggest that declines in animal and microbial decomposer diversity can slow leaf decomposition when exposed to toxic chemicals, whereas similar impairments by nutrient supply only occur at high enrichment levels (Beaumelle et al. 2020).

In addition to stressor type, the number of stressors—or stressor richness—may mediate the effects of biodiversity on ecosystem function, as observed in terrestrial ecosystems (Rillig et al. 2023; Song et al. 2023; Yang et al. 2022; Zhou et al. 2024). However, the number of stressors was confounded with overall stressor intensity in most of these studies (Schäfer et al. 2023), which may have masked effects of stressor richness on the biodiversity‐ecosystem function relationship (Holmes et al. 2021). This begs the question of whether increasing the number of stressors affects the BEF relationship even when the overall stressor intensity remains unchanged. In support of this idea, a theoretical analysis using three ecological community models found that stressor richness at fixed total stressor intensity levels increases the effect on ecosystem functions. By contrast, the effect on biodiversity was weaker and varied with the type of community model (Holmes et al. 2021). Empirical studies addressing this question are lacking.

**FIGURE 1:**
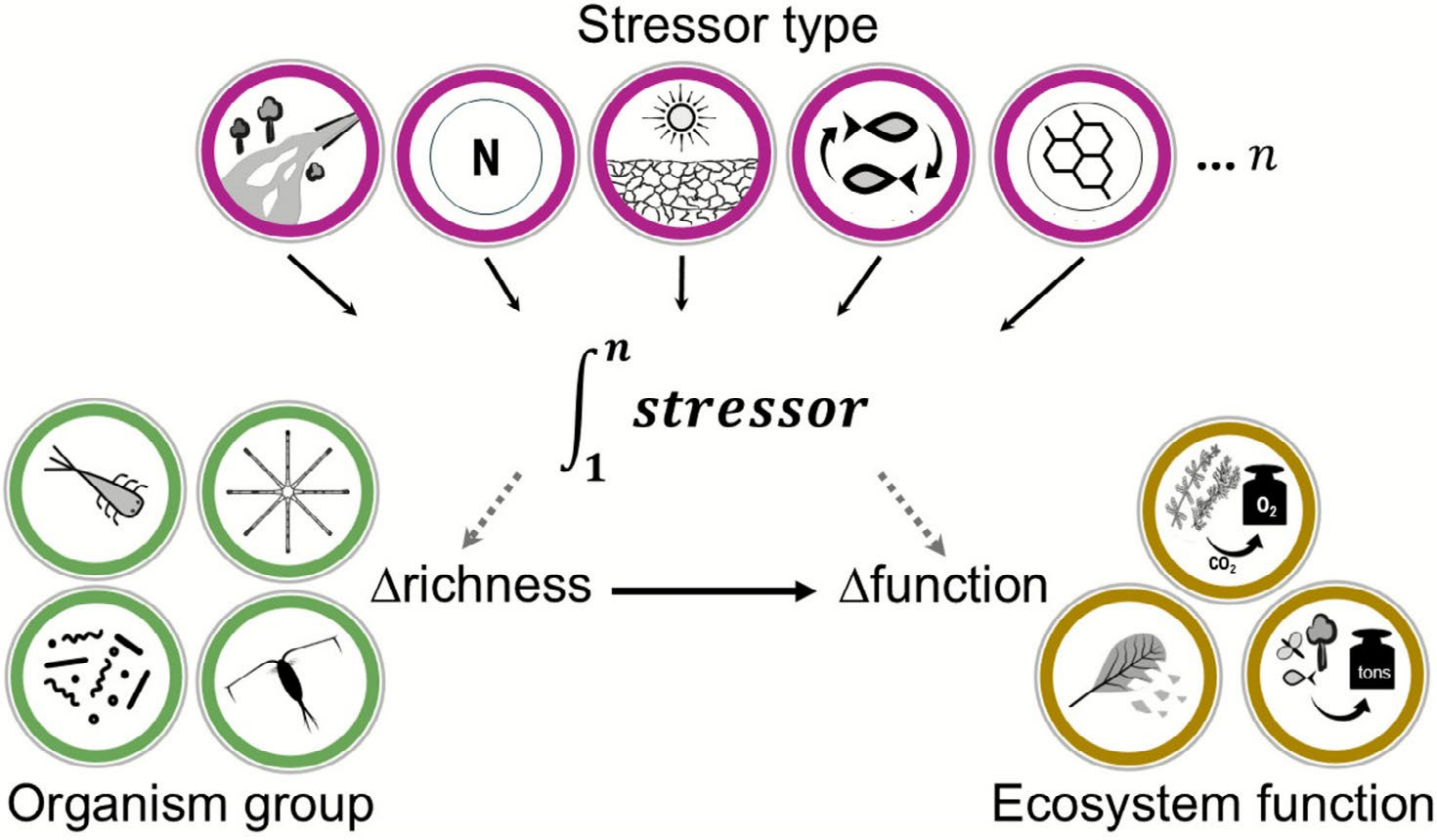
Overview of key questions addressed in the present meta‐analysis: how do the number and type of stressors, organism group and type of ecosystem function mediate relationships between changes in taxon richness and ecosystem function in freshwaters.

To address these knowledge gaps, we conducted a meta‐analysis to examine the effect of single and multiple stressor effects on the relationship between change in biodiversity (taxon richness) and change in ecosystem functions, and explored how the number of stressors, stressor type, organism group and type of ecosystem function affect this relationship (Figure 1). We hypothesised (1) that biodiversity loss driven by multiple stressors reduces ecosystem function (Beaumelle et al. 2020) and (2) that an increase in stressor richness exacerbates the effect (cf. Benkwitt et al. 2020). The latter hypothesis is based on the assumption that a combination of stressors differing in modes of action (e.g., physical vs. biochemical) increases the likelihood of losing dominant or keystone species that disproportionately affect ecosystem functions (Cardinale et al. 2012; Orr, Piggott, et al. 2024).

## 2 Methods

### 2.1 Data Selection for Meta‐Analysis

We queried a recently established database on multiple stressor effects in freshwaters (Orr, Macaulay, et al. 2024, Orr, Piggott, et al. 2024) for experimental studies that simultaneously determined responses of biodiversity and ecosystem functions. The search yielded 241 records, which we pre‐screened to assess whether both biodiversity and an ecosystem function were determined (Figure S1). We excluded 103 records lacking at least one piece of that information. Suitability of the remaining 138 records for the meta‐analysis was evaluated based on three criteria:

- Did the reported community comprise at least 4 taxa? Low taxon richness typically coincided with identifications at a high taxonomic level (e.g., phylum) and rarely resulted in a loss of taxa over the study period.
- Was an ecosystem function or a proxy of an ecosystem function determined? Accepted proxies included for production were rates of photosynthesis, enzyme activity and changes in biomass, in addition to two central ecosystem processes, primary production and organic matter decomposition (Table 1). Inclusion of proxies indicative of changes in ecosystem functions was facilitated by our choice to compare treatments with controls.
- Could the ecosystem function, or proxy thereof, be a priori attributed to the characterised community? While all freshwater communities can contribute to production of biomass, we specifically considered the contribution of primary producers to photosynthesis and of heterotrophic microorganisms and macroinvertebrates to litter decomposition (Table 1).

**TABLE 1:** Overview of broad organism groups and associated ecosystem functions or their proxies considered in the present meta‐analysis, with the number of individual observations and, in parentheses, the number of studies shown. Enzyme activity was excluded due to insufficient sample size and is therefore not shown.

Organismic group	Biomass^e^ (biomass/unit of volume or area)	Leaf decomposition (% mass loss/unit of time)	Photosynthesis^e^ (carbon produced/unit of volume or area)	Production (biomass produced/unit of time/volume or area)
Heterotrophic microorganisms^a^	3 (1)	62 (14)	0	10 (2)
Algae^b^	0	0	69 (12)	57 (9)
Zooplankton^c^	16 (4)	0	0	0
Macroinvertebrates^d^	0	81 (15)	0	0

^a^ Grouping of bacterial, fungal and other eukaryotic microbial communities.

^b^ Grouping of diatom, epilithic algal, periphytic and phytoplankton communities.

^c^ Grouping of micro‐ and metazooplankton communities.

^d^ Grouping of benthic macroinvertebrate communities.

^e^ Proxy of biomass production.

We excluded 60 of 138 records that failed to meet at least one of the three criteria (Table S1 and Figure S1). For 34 of the remaining 78 records, we extracted data on stressor treatments, taxon richness, Shannon diversity and ecosystem functions, along with general information such as geographical location and study duration.

To ensure consistency across the seven authors involved in data extraction, we provided written guidance and conducted an online training session using three test datasets. Discrepancies were discussed and used to refine the guidance. In addition, all extracted data were validated by one of two lead authors. For 44 of the remaining 78 records, the required information had been generated but not fully reported (Figure S1). We contacted the respective authors, who provided the requested data in 21 cases (Figure S1). Six records were omitted from the meta‐analysis for several reasons. For a study on macrophytes and two studies on enzyme activities, the effect size for a group could not be estimated with fewer than three data points. In another study, the experimental design deviated greatly from all others, hampering the calculation of a meaningful response metric. Finally, we removed two studies that only reported Shannon diversity and lacked data on taxon richness. Conversely, we added two of our own data sets that matched all of our criteria (see Text S1 for details). This resulted in a total of 51 records that were included in the meta‐analysis. Most records (85%) originated from short‐term (< 90 days) and small‐scale laboratory or mesocosm experiments (Table S1).

### 2.2 Data Pre‐Processing and Harmonisation

Data pre‐processing consisted of harmonising units and terminology by aggregating data to higher taxonomic or other grouping levels (i.e., heterotrophic microorganisms, algae, zooplankton, macroinvertebrates; Table 1), stressors (Table S2) and ecosystem functions (Table 1). This grouping mainly served to ensure a sufficient sample size for between‐group comparisons of taxa, stressors and functions, to balance sample size as much as possible, and to combine stressors inducing the same direction of effects. For example, we discriminated between stressors related to habitat (e.g., habitat disturbance, drought), nutrients and water quality, with the latter chiefly concerning contaminants, oxygen and temperature (Table S2). In studies that determined multiple functions per organism group, we considered only the combination of function and organism group that yielded the highest sample size across the database. For example, as only a few studies measured biomass of heterotrophic microorganisms and zooplankton besides leaf decomposition or biomass production, we prioritised the last two in the analyses. Most studies determined leaf decomposition and biomass production as ecosystem functions, as well as the rate of photosynthesis as a proxy for primary production (Table 1). In the 15 studies reporting data for multiple time points, we used the mean responses to aggregate data within each of those studies and thus harmonise the data set across all 51 studies, most of which reported only mean responses over time. Qualitatively similar results were obtained when using only the time point resulting in the greatest treatment effect in terms of taxon richness in a given study, defined as the time point where taxon richness in the treatment deviated most from the control (see Table S3 for details). All measurements made before exposure to stressors were omitted from the analyses.

### 2.3 Calculation of Log Response Ratios as a Measure of Effect Size

We used log response ratios (LRR) defined as the log (responsetreatment/responsecontrol) to quantify effect sizes and model the BEF relationship, with the control defined as in the original study, corresponding to the response without stressor exposure. Thus, we modelled the change in ecosystem function in response to a change in taxon richness or Shannon diversity. This approach is in line with other meta‐analyses examining how stressors influence the BEF relationship (Beaumelle et al. 2020) and allowed us to aggregate studies from diverse contexts by standardising treatment effect sizes as ratios relative to the control or baseline group (Hillebrand and Gurevitch 2016).

With small sample sizes, LRRs lead to a systematic over‐ or underestimation of effect sizes when the treatment mean is larger or smaller than the control mean, respectively (Lajeunesse 2015). This bias arises from increased sampling variability and can also affect variance estimates when parameter values are close to zero. To mitigate this bias, we applied the ‘all cases’ correction proposed by Nakagawa et al. (Nakagawa, Yang, et al. 2023; Nakagawa, Noble, et al. 2023), which introduces the smallest bias according to their comparative simulation study of correction methods. Approximately 1.3% of values were excluded because LRRs could not be calculated from zero or negative values. These cases arose from instances of zero taxon richness or ecosystem functions with negative production rates. Zero taxon richness occurred in two studies on leaf decomposition by aquatic fungi, where fungal presence was inferred from short‐term assays of aquatic hyphomycete sporulation. Negative production rates were associated with biomass loss under stressors. We removed only the specific data points; the studies were retained in the dataset.

### 2.4 Data Analysis

Prior to the data analysis, we harmonised all data across studies as follows. We invariably considered only the highest level of each stressor from a given study, because the majority of the considered studies (67%) included only two levels, namely one treatment level beside the control (Figure S2).

To test our hypotheses, we fitted a linear mixed‐effects model (LMM) with the LRR of ecosystem functions (LRREF) as the response variable, multiple predictors as fixed effects, and study ID as a random effect to account for between‐study differences. We included the LRR of taxon richness (LRRTR) as predictor as well as interactions with all other predictors to evaluate the BEF relationship and identify potential moderators. We also fitted a model using the LRR of Shannon diversity (LRRSD) instead of LRRTR, but given a considerably lower sample size and similar results, we primarily focus on LRRTR below. Additional predictors included the number of stressors (NS) as continuous predictors, and taxonomic group (TG), stressor combination (SC; e.g., nutrient, habitat, water quality; Figure 4) and type of ecosystem function (EF) as categorical predictors. Stressor combination was nested within number of stressors and taxonomic group within ecosystem function. Our model equation was:

$$\begin{array}{c} {\text{LRR}}_{\mathrm{EF}}\sim {\text{LRR}}_{\mathrm{TR}}+\mathrm{NS}/\mathrm{SC}+\mathrm{EF}/\mathrm{TG}+{\text{LRR}}_{\mathrm{TR}}\times \mathrm{NS}\\ \;+{\text{LRR}}_{\mathrm{TR}}\times \mathrm{EF}+{\text{LRR}}_{\mathrm{TR}}\times \mathrm{NS}/\mathrm{SC}+{\text{LRR}}_{\mathrm{TR}}\times \mathrm{EF}/\mathrm{TG} \end{array}$$

Under the assumption that stressor intensity is reflected by a change in taxon richness, the method we adopted allowed us to distinguish effects caused by the number of stressors and stressor intensity. This distinction was achieved by holding the change in taxon richness constant across different numbers of stressors considered in the model. In other words, we compared the effects of variation in the number of stressors on ecosystem functions while maintaining equivalent stressor intensities, as indicated by the same change in taxon richness. This approach is supported by the fact that results were similar when we replaced the log‐response ratio of taxon richness (LRRTR) by the log‐response ratio of Shannon diversity (LRRSD) in the model equation, which means also considering abundance changes (see Results). Thus, when adjusted for the observed change in taxonomic richness (aka stressor intensity), the effect of the number of stressors on ecosystem functions is unlikely to be merely driven by shifts in species abundance.

We tested for collinearity between predictors based on associations using Cramer's V and set a threshold for predictor pre‐selection at 0.7, in line with a simulation study (Dormann et al. 2013). All but the association between type of ecosystem function and taxonomic group (Cramer's *V* = 0.76) were below this threshold. Cramer's *V* was 0.18 and 0.17 for the association between stressor combination and type of ecosystem function and taxonomic group, respectively. However, this association was rather uninformative since both variables were nested factors in the model. We manually removed statistically non‐significant terms (*p* > 0.05) using stepwise backward selection to identify the most relevant predictors (Dunkler et al. 2014; Harrell 2015). For statistically significant interactions, the corresponding main effects were retained to ensure correct estimation and interpretation of the interaction terms (Finney 1948). We used Cook's distance to identify studies with a potentially disproportionate influence (values > 0.5, a commonly used conservative threshold) and refitted the model after removing those studies (Maindonald and Braun 2010). To further assess model robustness, we performed a leave‐one‐study‐out cross‐validation.

Publication bias was tested for a three‐level random‐effects meta‐analysis model using the *metafor* package (version 4.6.0) in R (Viechtbauer 2010). We used LRRTR and LRREF and their corresponding sampling variances as input variables. The model accounted for the hierarchical structure of the data, incorporating study‐level variance (between‐study heterogeneity) and within‐study variances (to account for multiple effect sizes reported in a given study). The restricted maximum likelihood (REML) method was used for parameter estimation. Subsequently, we used funnel plots and Egger's test to assess potential publication bias. In addition, we quantified heterogeneity at both the between‐ and within‐study level using the *I* ^2^ statistic (Nakagawa, Noble, et al. 2023).

We also calculated model deviation ratios (MDR) for both taxon richness and ecosystem function to identify non‐additive effects (i.e., synergism or antagonism). MDRs are commonly used in ecotoxicology for that purpose (Schäfer et al. 2023) and are defined as the ratio of observed effects to predicted effects based on single‐stressor impacts. Given that we used LRRs, additivity implies a multiplicative null model. This null model assumes a lack of an interaction between two given stressors and that the overall effect is the product of the independent probabilities (Schäfer and Piggott 2018). This means that for the loss of taxa from a community, the joint effect is the product of the individual proportional species losses. For the 16 studies involving more than two stressors, we only used stressor pairs in the analyses (i.e., stressor 1 and 2, stressor 1 and 3, and stressor 2 and 3) and omitted treatments with three or more stressors. We used the boundaries suggested in Cedergreen (2014), where MDRs > 2 are interpreted as synergism and MDRs < 0.5 are considered to be due to antagonism. For consistency, we multiplied LRRs by −1 when both stressors had negative signs, because otherwise the boundaries defined in Cedergreen (2014) for synergism and antagonism would be reversed. Moreover, we omitted 12 of 100 observations from the analysis where both LRRs were indistinguishable from noise around the control treatment and had opposite signs, because an analysis of those data would require a different definition of synergism and antagonism (Piggott et al. 2015). Noise was defined as ±25% of the global coefficient of variation around the control to balance the number of excluded cases with the inclusion of fairly small values around the control, which occasionally result in very high MDRs that are an artefact of dividing random numbers around the mean. Subsequently, we ran LMMs for both MDRs as described above to identify whether deviation from the null model depended on any of the predictors. Finally, we examined the association between both types of MDRs using Pearson correlations, to analyse whether deviations from the null model correlated between the LRRs of taxon richness and ecosystem functions.

All data were processed and analysed in R (version 4.4.2). The data and code are provided at https://doi.org/10.5281/zenodo.17545525.

## 3 Results

### 3.1 The Effect of Multiple Stressors on Biodiversity‐Ecosystem Function Relationship

**FIGURE 2:**
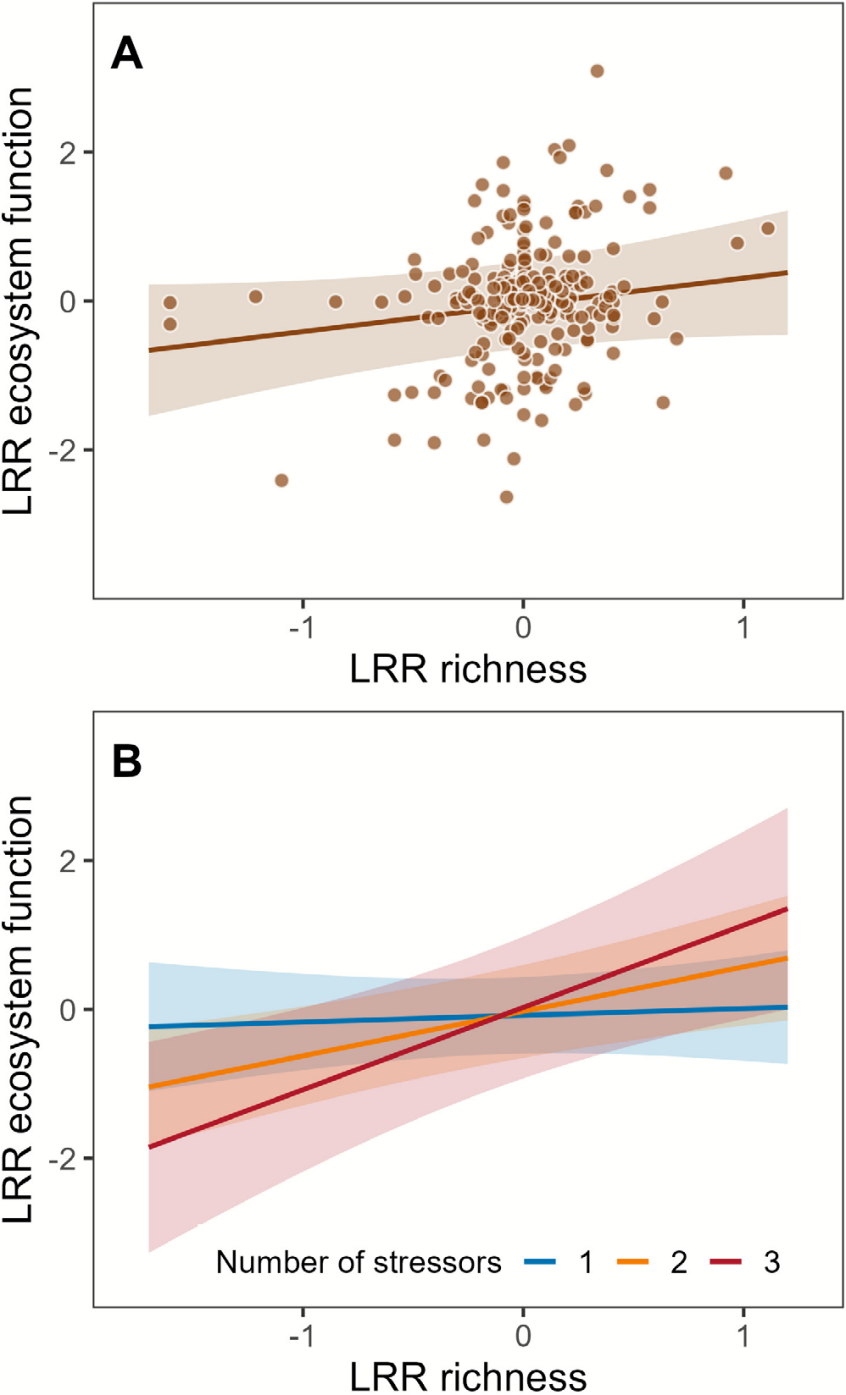
Marginal effect of (A) LRR richness on LRR ecosystem functions and (B) the interaction between LRR richness and number of stressors on LRR ecosystem functions (*p* = 0.02 in full model, Table S4). One outlier at *x* = 0.28 and *y* = −5.76 not shown. See Figure S3 for a version of panel B including the individual observations, which are not shown here for best visibility of trends. The line represents the predicted effect in the linear mixed‐effects model when all other variables are held constant. The shaded area represents the 95% confidence intervals of the model prediction.

**FIGURE 3:**
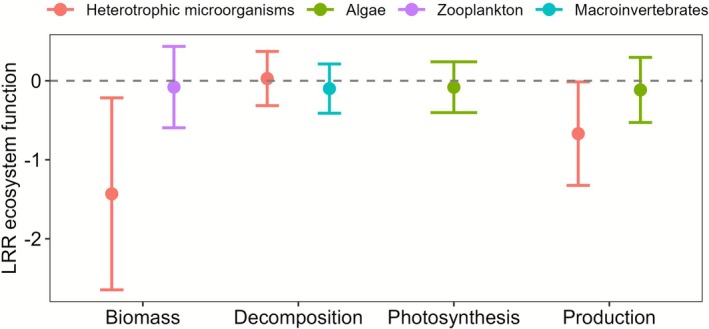
Marginal effect with 95% CI of organism group nested within type of ecosystem function on LRR ecosystem function (*p* = 0.04 in full model, Table S4, see Table S5 for individual parameters). Negative and positive values refer to decreased or increased ecosystem functions, respectively, in the treatment compared to the respective controls. See Tables 1 and S6 for sample sizes and Table S5 for details of the linear mixed‐effects model.

**FIGURE 4:**
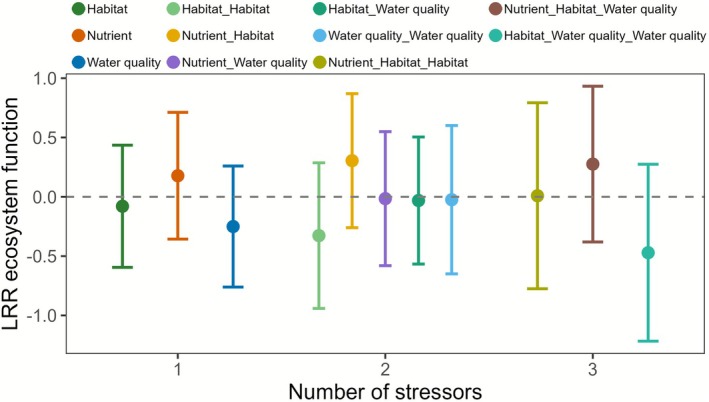
Marginal effect of the stressor combination nested within number of stressors on LRR ecosystem function (*p* = 0.03 in full model, Table S4, see Table S5 for individual parameters).Negative and positive values refer to decreased or increased ecosystem functions, respectively, in the treatment compared to the respective controls. Colours indicate the dominant stressor types: habitat (green), nutrients (orange), and water quality (blue). Combinations of stressors are shown as blends of these colours. See Table S5 for details of the linear mixed‐effects model and Table S6 for sample sizes.

The changes in taxon richness, which also reflect stressor intensity, and ecosystem functions covered a wide gradient ranging from approximately 0.2‐ and 0.1‐fold to 3‐ and 10‐fold of the controls, respectively (Figure 2). The best‐fit model after stepwise model selection contained the LRR of ecosystem function as the response variable and five explanatory variables (LRR richness, type of ecosystem function, number of stressors, organism group nested within type of ecosystem function, and stressor combination nested within number of stressors), as well as the interaction between LRR richness and number of stressors (linear mixed‐effects model: *n* = 271, 49 groups, marginal *R* ^2^ = 0.15, conditional *R* ^2^ = 0.44; see Table S4 for *p*‐values of variables). The large difference between the marginal and conditional *R* ^2^ points to a considerable between‐study variation, likely due to variation in study designs. Despite ample variation, the relationship between change in taxon richness and change in ecosystem functions was positive overall (Figure 2a). However, the number of stressors also influenced the relationship, indicated by a significant interaction with taxon richness (Figure 2b). Specifically, the slope of the relationship became steeper as the number of stressors increased from one to three, indicating that the effect of taxon loss on ecosystem function increases with the number of stressors. The model including change in Shannon diversity, accounting for abundances instead of taxon richness, displayed a very similar pattern (Figure S4). Neither the type of ecosystem function, organism group nor stressor combination significantly affected the BEF relationship (Table S4 and Figure S5). Despite variation among most organism groups and ecosystem functions, effect sizes were predominantly negative for the influence of heterotrophic microorganisms on microbial biomass and biomass production, revealing reductions in ecosystem functions (Figure 3). Furthermore, effects on functions related to productivity were more variable for heterotrophic microorganisms than for other organism groups. Combinations of stressors involving nutrient supply increased ecosystem functions more often than other combinations, although the difference was not statistically significantly (Figure 4). These stressor combinations also exhibited a tendency towards increased richness, but the pattern was less pronounced and consistent than for ecosystem functions (Figure S6). Removing two potentially influential studies identified by Cook's distance did not alter our two key results (Table S4), that is, neither the positive relationship between the LRRs of ecosystem function and taxon richness nor the significant interaction between LRR richness and number of stressors. Similarly, the leave‐one‐study‐out cross‐validation confirmed the robustness of the results (Table S5).

### 3.2 Predictability of Multiple‐Stressor Effects From Single Stressors

**FIGURE 5:**
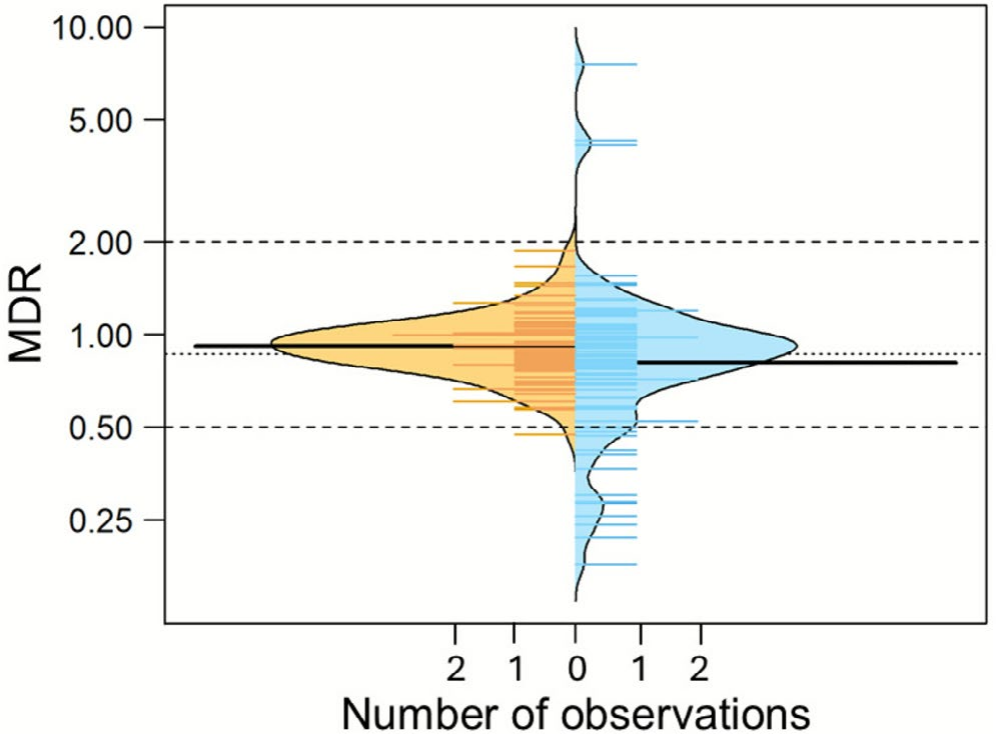
Bean plot of model deviation ratios (MDR) of the observed to predicted effect of multiple stressors for taxon richness (left, orange) and ecosystem function (right, light blue). Solid black lines represent the overall means, and coloured lines represent the individual values on which the modelled distribution is based. Longer lines indicate a higher number of observations. Dashed lines represent the MDR limits at 2 and 0.5, while the dotted line denotes the overall mean.

Observed effects of multiple stressors largely matched predictions based on the multiplicative null model that we adopted by using LRRs as a measure of effect size (Figure 5). With almost all MDRs falling within the conventional range of 0.5–2, indicative of additive effects (Cedergreen 2014), taxon richness did not display synergism, and only rarely antagonism (1%). MDRs for ecosystem function varied more strongly, including a few cases of synergism (3%) and a larger proportion of antagonistic effects (14%). The MDRs for taxon richness and ecosystem function were not correlated (*r* = −0.023, *p* = 0.83, *n* = 86). Neither taxonomic group, stressor combination nor type of ecosystem function significantly affected the MDRs for either taxon richness or ecosystem function (*p* > 0.17–0.65, *n* = 86, linear mixed‐effects models), except in the model for MDR ecosystem function where type of ecosystem function was close to statistical significance (*p* = 0.06, Figure S7). This suggests that deviation from the null model prediction, including synergism and antagonism, was largely independent of these predictors.

### 3.3 Assessment of Publication Bias

For taxon richness, neither the funnel plot nor Egger's test (*p* = 0.88) showed evidence of publication bias (Figure S8A). The funnel plot for ecosystem functions (Figure S8B) suggests a slight asymmetry towards positive effect sizes, but this was not significant (Egger's test, *p* = 0.30). Heterogeneity was primarily driven by between‐study variation, with *I* ^2^ values of 58% (taxon richness) and 84% (ecosystem function). Within‐study heterogeneity accounted for a much smaller proportion of variance (0% for taxon richness and 2% for ecosystem function). Significant heterogeneity beyond sampling error was detected with *Q* = 648 and 2762 for taxon richness and ecosystem functions, respectively (both df = 269 and *p* < 0.0001).

## 4 Discussion

The positive relationship we found between changes in taxon richness and changes in ecosystem function concurs with results of other meta‐analyses (Cardinale et al. 2012; Tilman et al. 2014). The increasing slope of the relationship with the number of stressors suggests that effects of biodiversity loss on ecosystem functions increase with the number of stressors, supporting our two initial hypotheses. A second key finding is that multiple‐stressor effects on both taxon richness and ecosystem functions largely followed predictions of our multiplicative null model, antagonistic effects on ecosystem functions constituting approximately 14% of casesnotwithstanding. We found no evidence that stressor type, type of ecosystem function or organism group influence the relationship between change in taxon richness and change in ecosystem function.

### 4.1 Relationship Between Biodiversity and Ecosystem Functions Under Multiple Stressors

Our finding that the slope of the relationship between change in taxon richness and change in ecosystem function increased with the number of stressors (Figure 2b) aligns with a theoretical study in which two of three alternative ecological community models demonstrated, at fixed stressor intensity, effects on ecosystem function increasing with the number of stressors (Holmes et al. 2021). Numerous empirical studies, predominantly from terrestrial ecosystems, have likewise reported concurrent declines in ecosystem functions and biodiversity as the number of stressors increases (Rillig et al. 2023; Song et al. 2023; Yang et al. 2022; Zhou et al. 2024). However, since overall stressor intensity generally increases with the number of stressors, these studies were unable to resolve the relative contributions of stressor richness versus intensity (Holmes et al. 2021; Schäfer et al. 2023). Our approach addresses this limitation by holding the change in taxon richness constant across different numbers of stressors, in contrast to previous studies, where taxon richness generally declined with stressor number. Assuming that stressor intensity is reflected in richness change, our method allowed us to disentangle the effects of stressor number from those of stressor intensity.

The greater impact on ecosystem function we observed at constant stressor intensity may result from a larger proportion of affected species in a community (Orr, Macaulay, et al. 2024; Orr, Piggott, et al. 2024). Unless correlations between species' tolerances to stressors are positive and strong, a phenomenon termed positive co‐tolerance, additional stressors affect a larger number of species (Holmes et al. 2021; Schäfer and Piggott 2018; Vinebrooke et al. 2004). Since stressors initially reduce the abundance of species before ultimately leading to species losses, an increase in the number of stressors is likely to lead to declines in the abundances of a larger fraction of species, even when overall taxon richness remains unchanged. These declines in abundance may in turn affect ecosystem functions. This explanation is consistent with the finding that our analysis using Shannon diversity, which reflects both taxon richness and evenness, produced similar results as the analysis based on taxon richness (Figure S4b). Therefore, even when evenness is maintained, the underlying distribution of abundances can shift to a larger proportion of affected species, disproportionately impacting functionally important taxa. Moreover, impacts of stressors may be sublethal, affecting the activity of species or altering species interactions, both of which can have consequences for ecosystem functions (Maltby and Hills 2008; Romanuk et al. 2010).

We found a positive relationship between changes in taxa richness and changes in ecosystem function consistent with the stressor gradient hypothesis (Bertness and Callaway 1994), which posits that positive interactions increase along an environmental stress gradient. However, this matching pattern does not necessarily imply the same underlying mechanism (Fugère et al. 2012). Indeed, our observed pattern could also result from additional stressors reducing functional redundancy, thereby leading to a closer coupling of biodiversity and ecosystem function as species are lost. A more detailed examination of the underlying mechanisms would require data on the functional traits of the organisms involved, which remain scarce for most organism groups included in our analysis.

### 4.2 Multiple‐Stressor Effects Match With Null Model Predictions

Multiple stressor effects on the relationship between taxon richness and ecosystem function largely followed the prediction of our multiplicative null model (Figure 5), although effects on ecosystem functions were antagonistic in about 14% of the relationships, that is, weaker than expected based on the individual effects. This aligns with an empirical study that identified predominantly multiplicative and antagonistic effects from 255 combinations of multiple chemical stressors (Smith et al. 2024). Our assessment is based on model deviation ratios (MDRs) along with defined MDR boundaries beyond which deviations from the null model prediction are considered synergistic or antagonistic effects. MDRs are commonly used in ecotoxicology to evaluate chemical mixture effects, whereas ecological studies have largely relied on statistical significance testing (Belden et al. 2007). Statistical significance testing, however, is strongly influenced by sample size and data variability and may therefore obscure between‐study comparisons (Popovic et al. 2024). Meta‐analyses on multiple‐stressor effects have often found relatively high frequencies of deviation from null model predictions, commonly between 50% and 80% of the considered cases, but the effect size of the deviations has only rarely been reported (Cote et al. 2016; Crain et al. 2008; Jackson et al. 2016). By contrast, using the conventional MDR cut‐offs of 0.5 and 2, only a few cases deviated from the null model prediction in our study, which took the shape of a normal probability distribution around the prediction (Schäfer et al. 2023). Thus, the majority of observed deviations may be driven by chance as opposed to an ecological mechanism.

The slightly skewed deviations towards antagonism observed for ecosystem functions (Figure 5) match a synthesis of meta‐analyses on multiple‐stressor effects, where the combined stressor effects at the community and ecosystem level were also predominantly antagonistic (Cote et al. 2016). In addition, a recent meta‐analysis of multiple stressor experiments found predominantly additive effects on leaf litter decomposition in freshwaters (Medina Madariaga et al. 2024). Several mechanisms may contribute to this dampening effect. First, compensatory dynamics of some taxa may increase their contribution to ecosystem functions in response to the decline or loss of other taxa, leading to a partial maintenance of ecosystem functions. Secondly, positive stressor co‐tolerances of taxa in conjunction with negative associations between tolerance and importance for maintaining ecosystem functions means that two given stressors would individually remove the same taxa that make notable contributions to ecosystem functions (Vinebrooke et al. 2004). When both of these stressors act jointly, the additional effect on ecosystem function would be disproportionately lower (i.e., less than additive = antagonistic). However, given the frequently missing information on community composition and the functional capacity of the constituent species, the significance of this mechanism remains speculative. This notwithstanding, our analysis revealed that two related ecosystem functions, biomass and biomass production, responded antagonistically (Figure S7), possibly suggesting compensatory dynamics driven by the persisting taxa that benefit from increased resource availability when sensitive taxa are lost. Such antagonistic effects may be limited to single ecosystem functions, yet disappear when responses of multiple ecosystem functions are assessed simultaneously in the context of multifunctionality (Li et al. 2024; Tilman et al. 2014). This could not be tested in our study due to a shortage of published data. Moreover, due to sample size constraints, our analysis was limited to pairs of stressors, although meta‐analyses have found non‐additive effects, particularly synergistic effects, to be more frequent as the number of stressors increases (Crain et al. 2008; Tekin et al. 2018).

In a nutshell, our analysis shows that while additional stressors exacerbate effects on ecosystem functions, even when overall stressor intensity is unchanged, this effect is largely predictable based on effects determined for individual stressors. However, despite this lack of synergistic effects, our findings highlight the urgency of mitigating biodiversity loss in the face of multiple stressors threatening species persistence with mostly additive effects on ecosystem functions.

### 4.3 Influence of Stressor and Organism Type on BEF Relationships

Neither stressor type, organism type nor another driver, apart from the number of stressors, affected BEF relationships, as indicated by the lack of interaction effects with LRR richness. This outcome contradicts previous meta‐analyses, which identified stressor type and type of ecosystem function as moderators of BEF relationships (Beaumelle et al. 2020; Cardinale et al. 2011; Hong et al. 2022). For example, biodiversity and leaf decomposition were significantly correlated under toxicant exposure but not under nutrient supply (Beaumelle et al. 2020). One potential explanation for this discrepancy is that variability in our dataset may have masked potential differences between stressors, because a two‐ to threefold lower sample size reduced statistical power. This explanation is at best partial, however, because another meta‐analysis involving a much larger sample size than the study by Beaumelle et al. (2020) also failed to detect differences between effects of nutrient supply and climatic stressors on BEF relationships (Hong et al. 2022). Generally, meta‐analyses of multiple stressors in freshwater ecosystems suggest that additive and synergistic community effects are common (Jackson et al. 2016; Morris et al. 2022). This observation points to frequent neutral and negative stressor co‐tolerances, suggesting that individual stressors often impact different species in freshwater communities. Consequently, unless all taxa contribute equally to an ecosystem function, different stressors are likely to have distinct effects on that function, underscoring the relevance of stressor type. However, for broad ecosystem functions such as community biomass production or organic matter decomposition where many, if not all taxa, contribute, these differences may be less pronounced, as observed in our study (Orr, Macaulay, et al. 2024; Orr, Piggott, et al. 2024).

Although stressor type did not affect the overall relationship between biodiversity and ecosystem function, our analysis revealed that exposure to stressors involving changes in nutrients produced a consistently positive mean response of ecosystem functions compared to control levels. This response differs to that of other stressors, which tended to show negative or variable mean responses (Figure 4), thus largely matching results of previous meta‐analyses. In particular, as noted above, Beaumelle et al. (2020) found predominantly negative effects for toxicants, whereas the direction of nutrient effects depended on the organism group, being predominantly negative for animals and positive for microbial decomposers. Likewise, Hong et al. (2022) reported mainly positive effects for nutrients, but generally negative or variable outcomes for temperature and drought. Lastly, a synthesis of responses to anthropogenic stressors also found consistently positive effects of nutrients on multiple ecosystem functions, whereas exposure to most other stressors yielded variable or negative responses (Brauns et al. 2022).

The negative response to stressors other than nutrients was expected because even when no species are lost, stressors can still reduce species abundances and impair physiological processes that are instrumental in ensuring ecosystem functions. Nutrients, in contrast, are a critical resource enabling biomass production, particularly by autotrophs. Nevertheless, nutrients can also impair organisms and ecosystem functions, whether indirectly, for example through oxygen depletion associated with lake eutrophication, or directly when nutrients or their transformation products, such as nitrite or ammonium, reach toxic levels (Berenzen et al. 2001). Accordingly, biodiversity, particularly of animals, showed a hump‐shape response to nutrients along a gradient across 71 lakes (Jeppesen et al. 2000), and a similar hump‐shaped relationship driven by the occurrence of benthic macroinvertebrates was observed on leaf decomposition across 100 headwater streams (Woodward et al. 2012), also underlined by the meta‐analysis of Beaumelle et al. (2020).

Finally, neither the type of ecosystem function nor the organism group, nested within ecosystem function type, influenced the BEF relationship in our study. This finding aligns with results of previous meta‐analyses by Hong et al. (2022) and Cardinale et al. (2011), who found that effects of taxon loss were similar for biomass production and nutrient uptake, which both showed amplifying negative responses to taxon loss (biomass production: 79%, nutrient uptake: 89%). However, only 61% of the studies examining organic matter decomposition followed this pattern. Despite these general trends, the high variability, small sample sizes per group and differences in gradient lengths of effect sizes related to taxon loss in our dataset may have masked potential differences between organism groups or ecosystem functions (Figure S5).

## 5 Conclusions

Our meta‐analysis provides empirical evidence that multiple stressors tend to exacerbate the effects of taxon loss on ecosystem function in freshwater ecosystems. The increasing steepness of the BEF relationship with the number of stressors rising illustrates that multiple stressors not only accelerate biodiversity loss but also amplify the functional consequences of a given level of loss in taxon richness or Shannon diversity, likely by disproportionately affecting functionally important taxa and altering community interactions. This underscores the need to consider multiple‐stressor impacts when assessing ecological responses to environmental change, irrespective of stressor type and taxonomic group. Our findings highlight the urgency of mitigating biodiversity loss in the face of multiple interacting stressors.

## Author Contributions

R.B.S., D.H., M.O.G., I.M.P., M.B., J.A.O.: conceptualisation. C.S., R.B.S., C.K.F., S.O.: validation. C.S., R.B.S., C.K.F., I.M.P.: formal analysis. D.B., A.J.B., S.A.B., J.B., M.O.G., M.B., A.B.‐C, B.J.C., G.M.D., A.F., P.F., U.H., T.T.Y.L., S.J.M., G.M.M., N.A.S.M., I.M.P., J.A.O., S.O., A.S., A.‐M.V.: data curation. R.B.S., C.S., M.V., S.A.B., M.W., B.S., H.S.B.: writing – original draft. All: writing – review and editing.

## Funding

This work was supported by Deutsche Forschungsgemeinschaft (426547801).

## Conflicts of Interest

The authors declare no conflicts of interest.

## Supporting information


**Data S1:** Supporting information.

**Table S1:** Supplementary table.

## Data Availability

The data and code are provided at https://doi.org/10.5281/zenodo.15178037.
