# Supplementary material for: Effects of Biodiversity Loss on Freshwater Ecosystem Functions Increase With the Number of Stressors
Source: Glob Chang Biol. 2025 Nov 26;31(11):e70617. doi: 10.1111/gcb.70617 (PMC12648459; doi:10.1111/gcb.70617)
Supplement: Supplementary file 1 — Data S1: Supporting information. [file GCB-31-e70617-s001.pdf]

Supplementary Information

Effects of biodiversity loss on freshwater ecosystem functions increase with the number of stressors

Authors: Ralf B. Schäfer[a], Daria Baikova, Helena S. Bayat, Arne J. Beermann, Stella A. Berger, Jens Boenigk, Mario Brauns, Andrea Burfeid-Castellanos, Bradley J. Cardinale, Gwendoline M. David, Alexander Feckler, Christian K. Feld, Patrick Fink, Mark O. Gessner, Una Hadziomerovic, Daniel Hering, T.T. Yen Le, Samuel J. Macaulay, Graciela Medina Madariaga, Ntambwe A. Serge Mayombo, Iris Madge Pimentel, James Orr, Stephen Osakpolor, Alexandra Schlenker, Bernd Sures, Anna-Maria Vermiert, Matthijs Vos, Markus Weitere, Christian Schürings[a]

[a] Both authors contributed equally

Text S1: Methods of two own studies included in the meta-analysis

Our analysis includes data from two stream mesocosm experiments that were unpublished at the time of our literature search. Although related studies have since been published (Madge Pimentel et al., 2024; Mayombo et al., 2024), we briefly outline the specific methods focussing on the included data sets below. We conducted two stream mesocosm experiments at the Boye River (latitude, longitude: 51.5533 °N, 6.9485 °E) using the *ExStream* setup (Piggott et al., 2015). We investigated stressor-induced changes in biotic communities of macroinvertebrates, aquatic hyphomycetes (fungi), and microalgae along with associated ecosystem functions, such as leaf decomposition and chlorophyll-*a* accumulation as a proxy of net primary production. Pumps continuously moved water from the adjacent stream into four header tanks on top of a scaffold, each supplying 16 circular mesocosms (outer diameter: 25 cm, central outflow: 6 cm, volume 3.5 L). This set-up provided environmentally realistic physico-chemical conditions and allowed entrainment of small organisms (< 5 mm) into the mesocosms. Each of the mesocosms received 1 L of sieved sediment, 200 mL of gravel, 3 stones, and 100 mL of a slurry of fine-particulate organic matter. Macroinvertebrate colonization was augmented by adding individuals collected by kick-net sampling at densities equivalent to approx. 0.05 m^2 of benthic stream habitat. We also distributed autumn-shed and air-dried alder leaves (*Alnus glutinosa*) enclosed in coarse-mesh (5 mm) and fine-mesh (0.5 mm) bags in the mesocosms to determine rates of leaf decomposition by macroinvertebrates and microorganisms, or by microorganisms only. The two experiments involved a three-week acclimation phase and a two-week stressor phase and were both started on March 4, 2022.

The first experiment addressed impacts of salinisation (ambient vs. average of +150 mg/L chloride), flow velocity reduction (14.2 vs. 3.5 cm/s) and warming (ambient vs. average of +3.5 °C) in a full-factorial design. In the second experiment, we investigated effects of salinisation (ambient vs. +136 mg/L chloride), warming (ambient vs. +3.4 °C) and predator exposure, the latter in accordance with Germany's Animal Welfare Act. Three-spined sticklebacks (*Gasterosteus aculeatus*; size 4.0-7.0 cm) and bullheads (*Cottus rhenanus*; size 3.8-7.2 cm) were evenly distributed between two fish tanks (604 L, total fish biomass of 480g) and fed daily with invertebrates. Water from both tanks was pumped into an irrigation line, diluted and distributed to the mesocosms to expose the invertebrates to fish and alarm cues (final fish biomass of 0.005 g/L; 0.8 % of the predator cues in the mesocosms). In addition, each of the mesocosms exposed to these chemical cues received one bullhead starting five days after stressor exposure.

At the end of the stressor phase, we sampled all mesocosms, 32 per experiment, for macroinvertebrates, microalgae and fungi and determined rates of leaf decomposition and chlorophyll-*a* contents on the mesocosm walls. Macroinvertebrates were separated from the decomposing leaf material and stored in 96% ethanol. Microalgal communities were scraped off the mesocosm walls (100 cm^2 areas) and also preserved in 96% ethanol. For fungal communities, leaf discs were incubated for 24 h to obtain spore suspensions, which we preserved in formalin (2% final concentration). We used CO1 (primers: fwhF2/fwhR2n)(Vamos et al., 2017) and 18S-V9 amplicon sequencing (primers: 1389F/1510R) (Amaral-Zettler et al., 2009) for the homogenised macroinvertebrate tissue and periphyton to determine community composition. Operational Taxonomic Units (OTU) were used as a proxy for species. Fungal spores were identified and counted under a microscope (x 200). Ergosterol was used as a proxy for fungal biomass (Gessner, 2020). The leaf material remaining in litter bags was freeze-dried and weighed to the nearest 0.01 g

to calculate leaf mass loss relative to leached control leaves. Chlorophyll-*a* content as a proxy for primary producer biomass of periphyton acclimated to dark conditions was estimated fluorometrically using a BenthoTorch (BBE+ Moldaenke) and a MINI-PAM II fluorometer (Heinz Walz GmbH). For all biological communities, we determined taxon richness. After completing the experiment, the fish were returned to their respective collection area.

Table S2: Stressor classification in three groups.

Stressor group	Stressor type in original study
Habitat conditions	Absence of artificial plants, streambed disturbance, flow alteration, fine sediment input, reduction in ultraviolet radiation, water turbulence, light reduction, drought, change in rainfall, infection, predation
Nutrients	Nutrient supply
Water quality	Acidification, metal exposure, nanoparticle exposure, sulfur exposure, oxygen decline, salinisation, CO_2 increase, temperature change, exposure to antimicrobials, insecticide exposure, micropollutant exposure, pesticide exposure

Table S3: Influence of two different methods used to aggregate multiple time points during data pre-processing on results of the present meta-analysis.

Variables and results[a]	Mean richness and ecosystem functions over time points[b]	Time point of maximum richness difference to control[b]
	P-values for variables in model explaining LRR Ecosystem function (LRR_{EF})	
Main effect LRR richness (LRR_{TR})	**p = 0.02**	p = 0.06
Interaction of LRR richness (LRR_{TR}) and number of stressors	**p = 0.02**	**p = 0.01**
Taxonomic group nested within ecosystem function	**p = 0.04**	**p = 0.03**
Interaction of number of stressors and stressor combinations	**p = 0.03**	**p = 0.01**
	Fraction of cases outside MDR range (i.e. >2 or <0.5) for richness	
MDR > 2	0 %	0 %
MDR < 0.5	1.2 % (1 observation)	2.4 % (2 observations)
	Fraction of cases outside MDR range (i.e. >2 or <0.5) for ecosystem functions	
MDR > 2	3.3 % (3 observations)	4.9 % (4 observations)
MDR < 0.5	14.1 % (13 observations)	13.5 % (11 observations)
	P-values for variables in the model explaining MDR Ecosystem function	
Effect of type of ecosystem function	p = 0.06	**p = 0.04**
	P-values for variables in the model explaining MDR Richness	
Effect of taxonomic group	p = 0.10	p = 0.30
Correlation between MDR Richness and MDR Ecosystem function	r = -0.023; p = 0.83	r = -0.15; p = 0.19

[a] MDR = Model deviation ratio. LRR = log response ratio.

[b] See main paper for details of the approach.

Table S4: Analysis of Deviance (Type II Wald χ^2 tests) for fixed effects in linear mixed-effects models resulting from manual backward elimination of terms and when removing influential observations from the model based on Cook's distance. Nested factors are indicated by a slash (/), where the factor following the slash is nested within the higher-level factor preceding it. Interactions are indicated by a cross multiplication sign (×). The non-significant variables Ecosystem function type and Number of stressors were were retained in the model because they contributed to significant nested factors or interactions. See Table S5 for parameter estimates.

Term	Model			Model excluding two influential studies		
Predictor[a]	χ^2	df[b]	p-value	χ^2	df[b]	p-value
LRR taxon richness (LRR_{TR})	5.6	1	**0.02**	3.1	1	0.07
Ecosystem function type	1.0	3	0.79	1.2	3	0.74
Number of stressors	0.8	1	0.35	1.0	1	0.32
Ecosystem function type / Taxonomic group	8.1	3	**0.04**	11.4	3	**0.01**
Number of stressors / Stressor combination	18.2	9	**0.03**	23.0	9	**0.01**
LRR taxon richness (LRR_{TR}) × Number of stressors	5.0	1	**0.02**	4.3	1	**0.03**

[a]LRR = log response ratio.

[b]df = degrees of freedom.

Table S5: Parameter estimates for fixed effects in the linear mixed-effects model resulting from manual backward elimination of terms (see Table S4 for the significance of predictors) and leave-one-study-out cross-validation (LOSO CV). The regression coefficients for the categorical variables show the deviation of the respective level from a baseline given by the intercept when all other variables are held constant. The intercept represents the following levels of the categorical variables: biomass for the ecosystem functions, the alternative taxonomic group to heterotrophic microorganisms nested within the respective ecosystem function (Figure 3; i.e. zooplankton, macroinvertebrates and algae) and habitat for stressor combination nested within the number of stressors (Figure 4). Nested factors are indicated by a slash (/), where the factor following the slash is nested within the higher-level factor preceding it. Interactions are indicated by a multiplication sign (×).

Term[a]	Coefficient estimate	LOSO CV			Standard error	p-value
		Median	2.5%	97.5%		
Intercept	-0.13	-0.13	-0.22	-0.01	0.37	0.72
LRR richness (LRR_{TR})	-0.42	-0.42	-0.56	-0.32	0.40	0.30
EF leaf decomposition	-0.02	-0.02	-0.12	0.03	0.25	0.93
EF photosynthesis	0.00	0.00	-0.11	0.06	0.27	0.99
EF production	-0.04	-0.04	-0.15	0.12	0.31	0.91
Number of stressors	0.05	0.05	0.01	0.12	0.23	0.81
EF biomass / TG heterotrophic microorganisms	-1.35	-1.35	-1.46	-1.32	0.65	**0.04**
EF leaf decomposition / TG heterotrophic microorganisms	0.13	0.13	0.07	0.18	0.18	0.49
EF production / TG heterotrophic microorganisms	-0.55	-0.55	-0.59	-0.52	0.31	0.08
Number of stressors / SC habitat + habitat	-0.15	-0.15	-0.19	-0.11	0.14	0.29
Number of stressors / SC habitat + water quality	0.00	0.00	-0.03	0.02	0.11	0.98
Number of stressors / SC habitat + water quality + water quality	-0.17	-0.17	-0.20	-0.12	0.16	0.29
Number of stressors / SC nutrients	0.26	0.26	0.23	0.30	0.16	0.10
Number of stressors / SC nutrients + habitat	0.16	0.17	0.13	0.20	0.13	0.20
Number of stressors / SC nutrients + habitat+habitat	-0.01	-0.01	-0.06	0.04	0.18	0.97
Number of stressors / SC nutrients + habitat + water quality	0.08	0.08	0.03	0.13	0.16	0.61
Number of stressors / SC nutrients + water quality	0.00	0.00	-0.03	0.04	0.13	0.97
Number of stressors / SC water quality	-0.17	-0.17	-0.19	-0.14	0.14	0.24
LRR richness × number of stressors	0.51	0.51	0.44	0.60	0.23	**0.03**

[a] LRR = log response ratio, EF = ecosystem function, TG = taxonomic group, SC = stressor combination.

Table S6: Overview of combinations of broad stressor types used in the individual studies considered for the present meta-analysis. Combinations of stressor types with less than 5 observations were excluded from the analysis.

Combination of stressor types	Number of individual observations	Number of cases			
		Algae	Hetero-trophic micro-organisms	Macro-inverte-brates	Zoo-plankton
Habitat	52	21	10	18	3
Nutrients	39	21	8	8	2
Water quality	61	23	22	12	4
Habitat + habitat	17	9	4	4	0
Habitat + water quality	31	10	7	11	3
Nutrients + habitat	23	11	3	8	1
Nutrients + nutrients	3	3	0	0	0
Nutrients + water quality	21	7	7	5	2
Water quality + water quality	14	6	7	1	1
Habitat + habitat + habitat	2	1	1	0	0
Habitat + water quality + water quality	6	3	2	1	0
Nutrients + habitat + habitat	5	3	1	1	0
Nutrients + habitat + water quality	10	4	1	4	1
Nutrients + water quality + water quality	1	0	1	0	0
Water quality + water quality + water quality	1	1	0	0	0

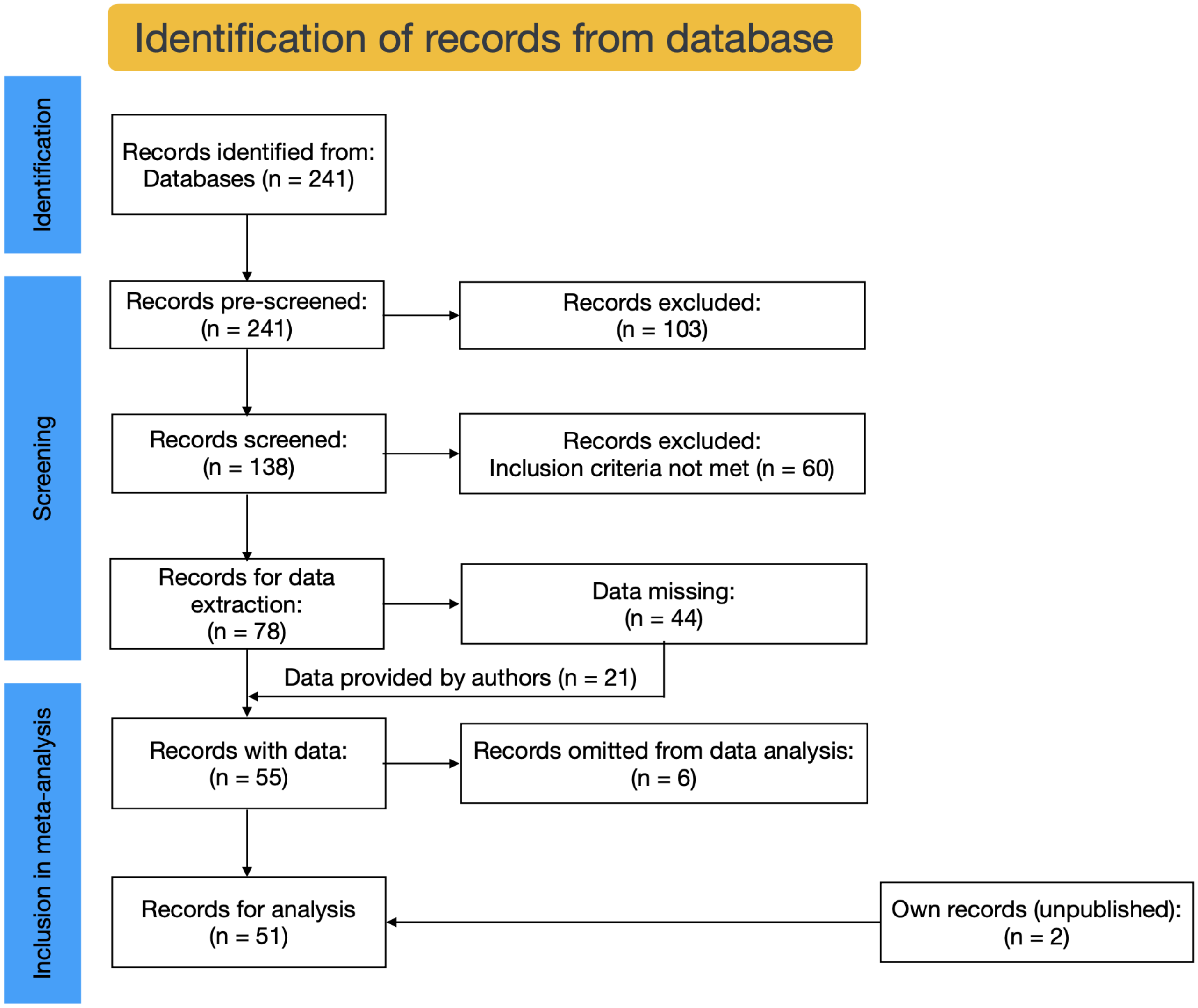

Figure S1: PRISMA flow chart illustrating the sequential selection of records for the present meta-analysis.

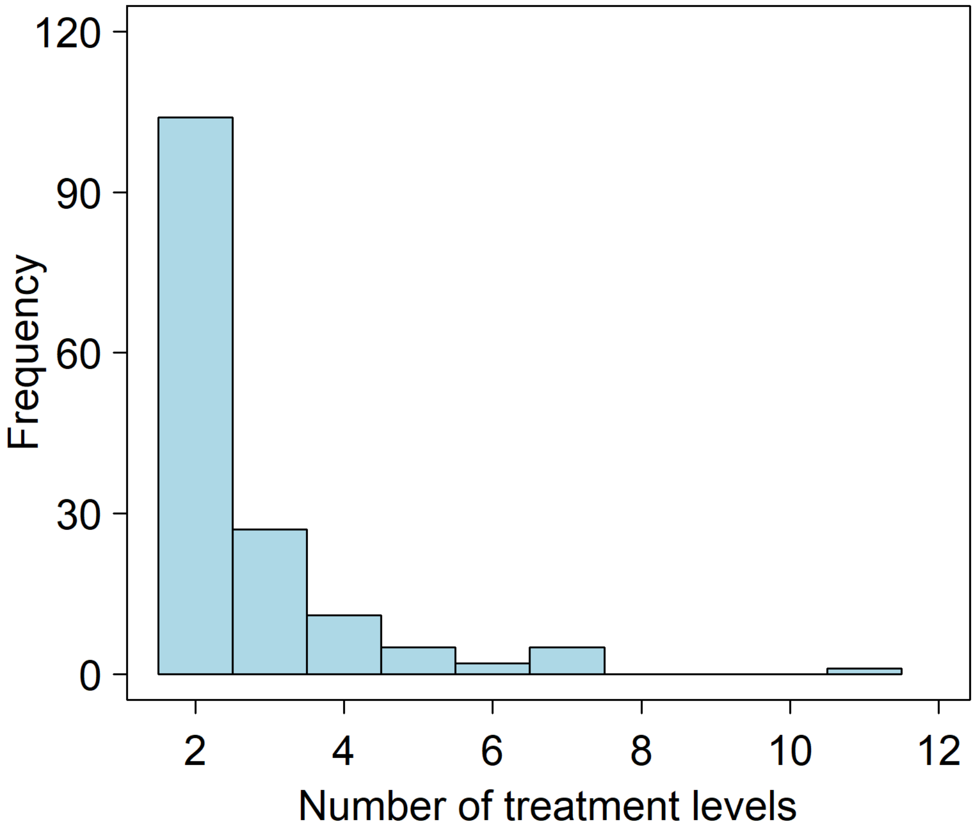

Figure S2: Number of treatment levels in the studies included in the present analysis.

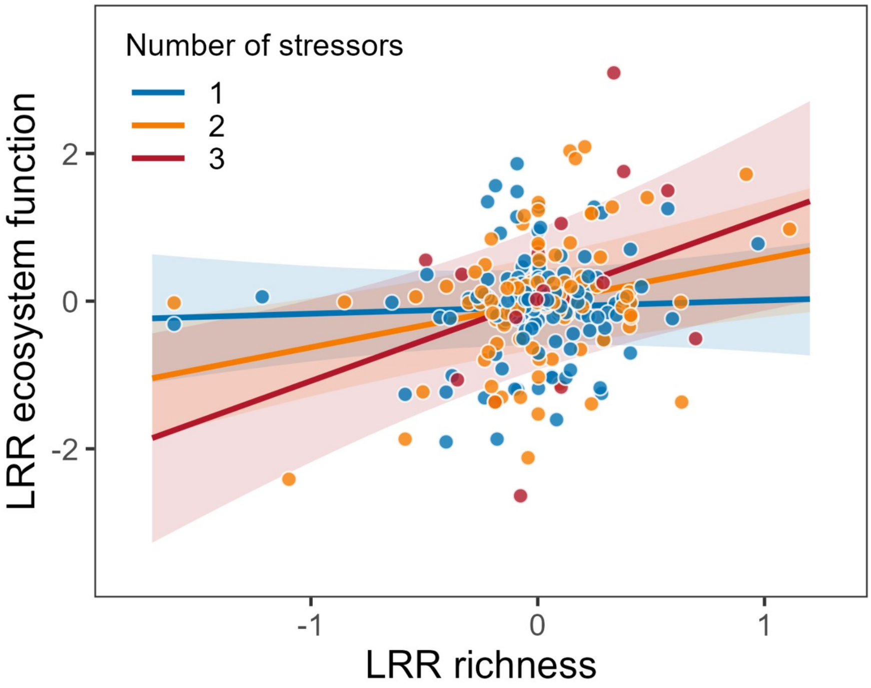

Figure S3: Relationships between LRR taxon richness and LRR ecosystem functions in the presence of one, two or three stressors. One outlier at x = 0.28 and y = -5.76 not shown.

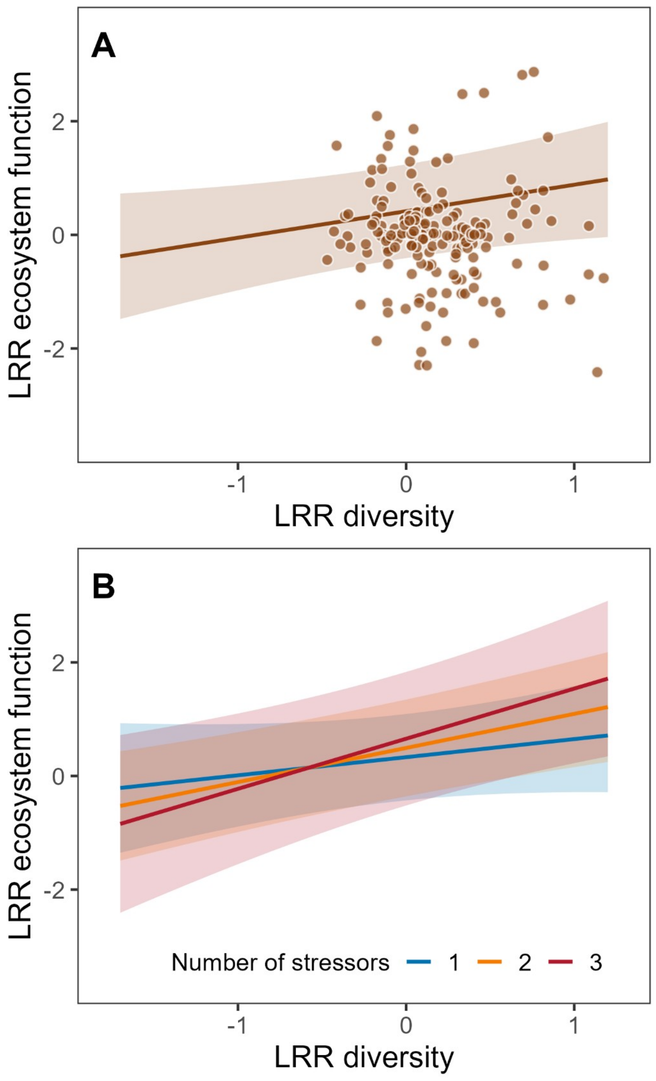

Figure S4: Marginal effects of A) LRR Shannon diversity on LRR ecosystem functions and B) the interaction between LRR Shannon diversity and the number of stressors on LRR ecosystem functions. Three outliers (-2.65, -2.41), (1.43, 2.67) and (1.37, 2.30) not shown in panel A. Lines in panel B represent the predicted effects in the linear mixed-effects model when all other variables are held constant. The shaded areas represent 95% confidence intervals of the model predictions.

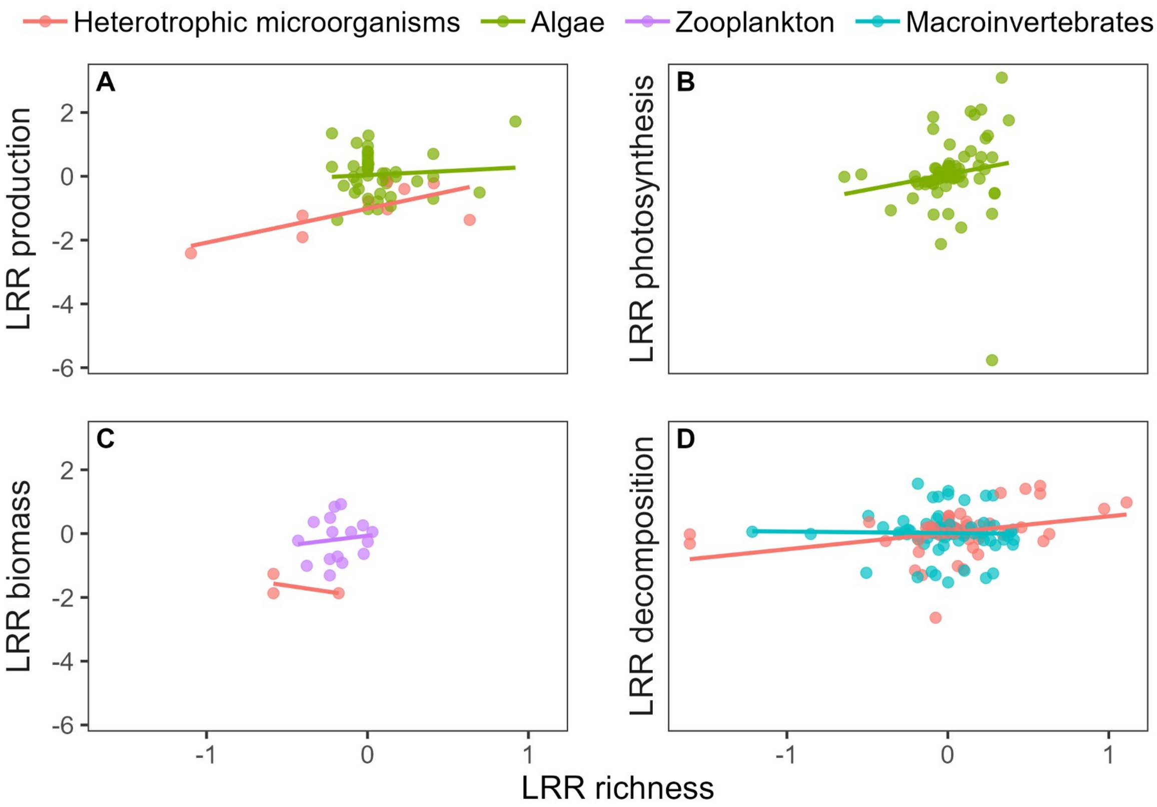

Figure S5: Relationship between LRR ecosystem function and LRR richness for the four different ecosystem functions and organism groups considered in the present meta-analysis.

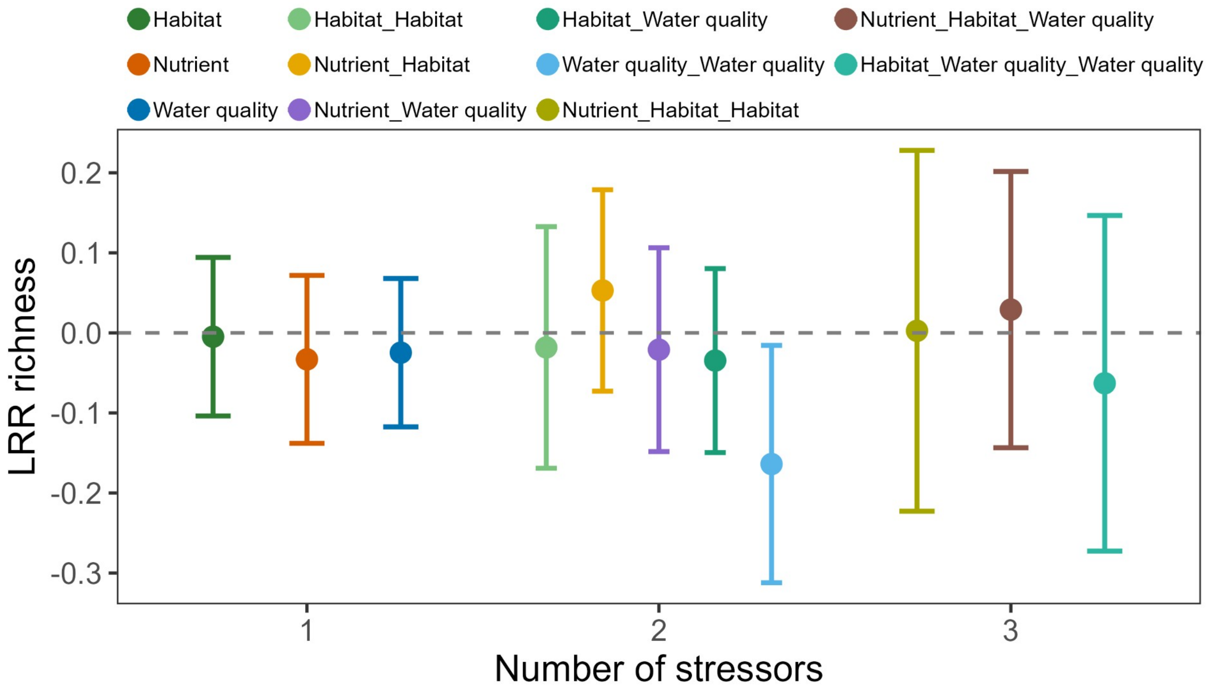

Figure S6: Marginal effects of stressor combination nested within number of stressors on LRR richness. Values below and above 0 mean that richness in the treatment was decreased or increased, respectively, compared to the respective controls. See Table S6 for sample sizes.

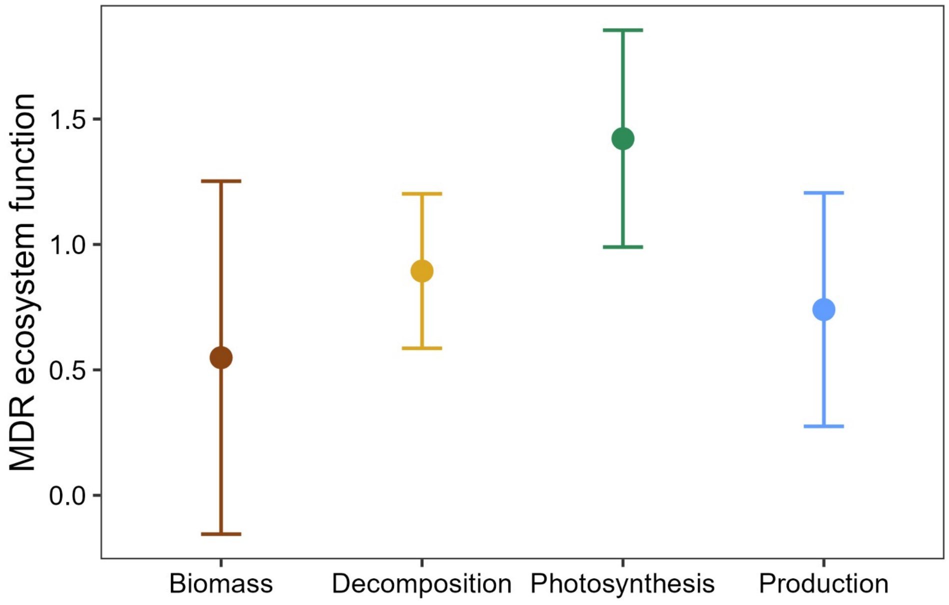

Figure S7: Predicted effect of type of ecosystem function on the model deviation ratio (MDR) for ecosystem functions. Values below and above 1 denote whether MDRs leaned towards antagonism or synergism, respectively. Linear mixed-effects model: n = 99, 44 groups, marginal R^2 = 0.09; conditional R^2 = 0.20.

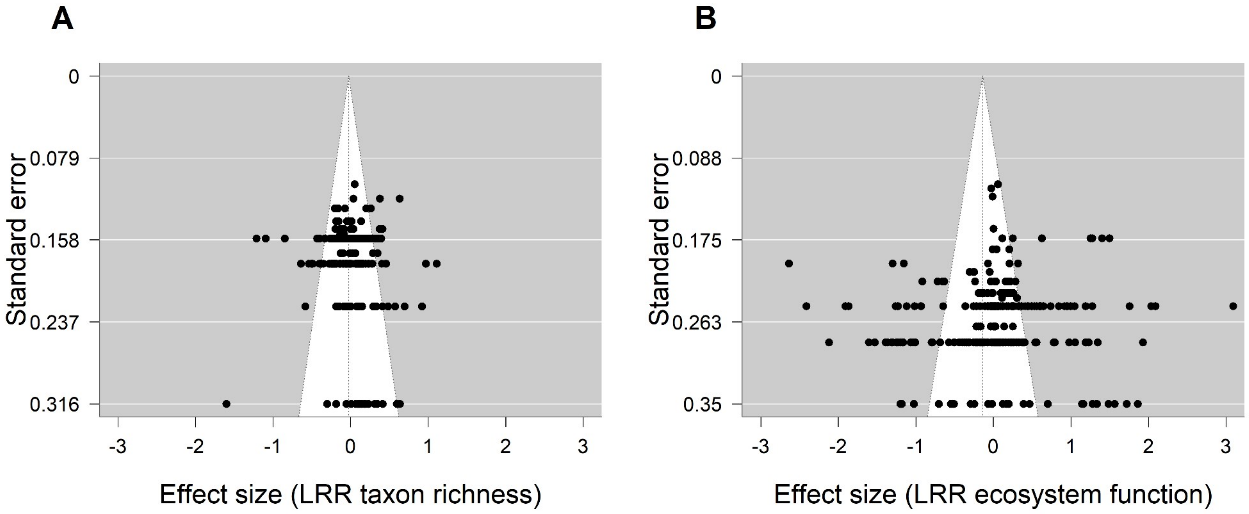

Figure S8: Funnel plots for A) LRR taxon richness and B) LRR ecosystem function. The absence of a clear asymmetry of effect sizes and Egger's test indicate a publication bias neither for taxon richness (p = 0.88) nor for ecosystem function (p = 0.30).

References

Amaral-Zettler, L. A., McCliment, E. A., Ducklow, H. W., & Huse, S. M. (2009). A Method for Studying Protistan Diversity Using Massively Parallel Sequencing of V9 Hypervariable Regions of Small-Subunit Ribosomal RNA Genes. *PLoS ONE, 4*(7), e6372. https://doi.org/10.1371/journal.pone.0006372

Gessner, M. O. (2020). Ergosterol as a Measure of Fungal Biomass. In F. Bärlocher, M. O. Gessner, & M. A. S. Graca (Eds.), *Methods to Study Litter Decomposition: A Practical Guide* (pp. 247–255). Springer International Publishing. https://doi.org/10.1007/978-3-030-30515-4_27

Madge Pimentel, I., Baikova, D., Buchner, D., Burfeid Castellanos, A., David, G. M., Deep, A., Doliwa, A., Hadžiomerović, U., Mayombo, N. A. S., Prati, S., Spyra, M. A., Vermiert, A.-M., Beisser, D., Dunthorn, M., Piggott, J. J., Sures, B., Tiegs, S. D., Leese, F., & Beermann, A. J. (2024). Assessing the response of an urban stream ecosystem to salinization under different flow regimes. *Science of The Total Environment, 926*, 171849. https://doi.org/10.1016/j.scitotenv.2024.171849

Mayombo, N. A. S., Burfeid-Castellanos, A. M., Vermiert, A.-M., Pimentel, I. M., Rehsen, P. M., Dani, M., Jasinski, C., Spyra, M. A., Kloster, M., Vidaković, D., Buchner, D., & Beszteri, B.

(2024). Functional and compositional responses of stream microphytobenthic communities to multiple stressors increase and release in a mesocosm experiment. *Science of The Total Environment*, *943*, 173670. https://doi.org/10.1016/j.scitotenv.2024.173670

Piggott, J. J., Townsend, C. R., & Matthaei, C. D. (2015). Climate warming and agricultural stressors interact to determine stream macroinvertebrate community dynamics. *Global Change Biology*, *21*(5), 1887–1906. https://doi.org/10.1111/gcb.12861

Vamos, E. E., Elbrecht, V., & Leese, F. (2017). Short COI markers for freshwater macroinvertebrate metabarcoding. *Metabarcoding and Metagenomics*, *1*, e14625. https://doi.org/10.3897/mbmg.1.14625
